# The OnTrack Diabetes Web-Based Program for Type 2 Diabetes and Dysphoria Self-Management: A Randomized Controlled Trial Protocol

**DOI:** 10.2196/resprot.2813

**Published:** 2015-08-04

**Authors:** Mandy Cassimatis, David John Kavanagh, Andrew Paul Hills, Anthony Carl Smith, Paul A Scuffham, Christian Gericke, Sophie Parham

**Affiliations:** ^1^ The Wesley Health and Medical Research Centre Rural and remote health centre Brisbane Australia; ^2^ Queensland University of Technology Institute of Health and Biomedical Innovation Brisbane Australia; ^3^ Mater Mothers Hospital, Mater Medical Research Institute Level 3 Aubigny Place, Raymond Terrace South Brisbane, 4101 Brisbane Australia; ^4^ Griffith Health Institute Griffith University Brisbane Australia; ^5^ University of Queensland Centre for Online Health Ground Floor, Main Building, Princess Alexandra Hospital Woollongabba, 4102 Brisbane Australia; ^6^ Griffith University School of Medicine & Griffith Health Institute Griffith University Drive Meadowbrook, 4131 Brisbane Australia; ^7^ The Wesley Health and Medical Research Centre Level 8 East Wing, The Wesley Hospital Auchenflower, 4066 Brisbane Australia

**Keywords:** diabetes mellitus, Type 2, depression, anxiety, self care, Internet, Web, online systems, intervention studies, randomized controlled trial, therapy, computer-assisted

## Abstract

**Background:**

The prevalence of type 2 diabetes is rising with the majority of patients practicing inadequate disease self-management. Depression, anxiety, and diabetes-specific distress present motivational challenges to adequate self-care. Health systems globally struggle to deliver routine services that are accessible to the entire population, in particular in rural areas. Web-based diabetes self-management interventions can provide frequent, accessible support regardless of time and location

**Objective:**

This paper describes the protocol of an Australian national randomized controlled trial (RCT) of the OnTrack Diabetes program, an automated, interactive, self-guided Web program aimed to improve glycemic control, diabetes self-care, and dysphoria symptoms in type 2 diabetes patients.

**Methods:**

A small pilot trial is conducted that primarily tests program functionality, efficacy, and user acceptability and satisfaction. This is followed by the main RCT, which compares 3 treatments: (1) delayed program access: usual diabetes care for 3 months postbaseline followed by access to the full OnTrack Diabetes program; (2) immediate program: full access to the self-guided program from baseline onward; and (3) immediate program plus therapist support via Functional Imagery Training (FIT). Measures are administered at baseline and at 3, 6, and 12 months postbaseline. Primary outcomes are diabetes self-care behaviors (physical activity participation, diet, medication adherence, and blood glucose monitoring), glycated hemoglobin A1c (HbA1c) level, and diabetes-specific distress. Secondary outcomes are depression, anxiety, self-efficacy and adherence, and quality of life. Exposure data in terms of program uptake, use, time on each page, and program completion, as well as implementation feasibility will be conducted.

**Results:**

This trial is currently underway with funding support from the Wesley Research Institute in Brisbane, Australia.

**Conclusions:**

This is the first known trial of an automated, self-guided, Web-based support program that uses a holistic approach in targeting both type 2 diabetes self-management and dysphoria. Findings will inform the feasibility of implementing such a program on an ongoing basis, including in rural and regional locations.

**Trial Registration:**

Australian and New Zealand Clinical Trials Registration number: ACTRN12612000620820; https://anzctr.org.au/Trial/Registration/TrialReview.aspx?ACTRN=12612000620820 (Archived by WebCite at http://www.webcitation.org/6a3BeXC5m).

## Introduction

Diabetes mellitus affects an estimated 346 million people globally and type 2 diabetes accounts for 85% to 90% of all cases [1]. In Australia, type 2 diabetes affects 4% of the population [2]. Diabetes constitutes the eighth-highest burden of disease in Australia and type 2 diabetes accounts for 92% of this burden [3]. With the rapidly rising prevalence of diabetes, the effects of inadequate diabetes self-management continue to increase and resulting diabetes complications and premature mortality become more urgent to address. Diabetes is the leading cause of blindness, end-stage renal disease, and lower limb amputation in the world [1]. Moreover, the effects of diabetes on quality of life, mental health, work productivity, and other intangible losses are substantial and add further to the diabetes-related burden to society.

Depression [4,5] and anxiety [6] are significantly more prevalent in people with diabetes than in the general population. Comorbid mental health conditions are barriers to effective diabetes self-care [7] and further increase diabetes-related expenditures [8]. Dysphoria complicates the achievement of adequate glycemic control through multiple pathways. Comorbid depression and/or anxiety with diabetes predisposes individuals to diabetes-specific distress [9,10] and affects glycemic control [11] both directly (via physiological mechanisms [8-10]) and indirectly by reducing motivation for adequate self-management [12-14]. Even subclinical manifestations, such as dysphoria, are associated with clinically deleterious outcomes, including reduced self-care [7], poorer glycemic control [15,16], increased incidence and progression of diabetes complications [17,18], and greater disability [19]. Stress contributes to other common physical comorbidities in diabetes patients, including metabolic syndrome and cardiovascular disease [20]. Diabetes-specific distress (DSD)—the emotional and psychological burden posed by diabetes [21]—accounts for a high proportion of variance in depression [22] and further impedes self-management [23]. Combined effects of inadequate glycemic control and dysphoric mood in diabetes [21,22] thus call for urgent intervention.

Prior research indicates the need for behavioral diabetes self-management interventions to incorporate psychological and emotional support components for optimum efficacy [23]. Improvements in diabetes self-care are strongly associated with improved glycemic control [24,25], so behavioral interventions to support diabetes self-care might also improve clinical outcomes [26]. However, improvements in glycemic control do not generally result in significant improvements in mood [23], nor do psychological treatments for depression and anxiety in people with diabetes reliably produce improvements in glycemic control [27]. Despite the limited impact of single-focused interventions in diabetes [28], self-management interventions typically fail to address both issues. Furthermore, trials on self-management interventions often only follow-up participants for relatively short periods (eg, ≤6 months [29]) so that long-term effects remain undetermined.

There are many barriers to adequate treatment of type 2 diabetes within health systems, including a shortage of health professionals, inadequate availability of services, limited access in rural and remote regions, practitioner/patient communication problems, time pressures in medical consultations, and limitations in the skills and confidence of health professionals in the provision of psychological interventions [30]. Patients often struggle to manage the complexity of type 2 diabetes treatment regimens [31], to recognize dysphoria symptoms [32,33], acknowledge their need for support, and find the motivation to overcome barriers to a healthy lifestyle [34]. Shortcomings in health care services particularly affect patients at risk of psychological comorbidities and the number of these patients will escalate sharply as the incidence of diabetes increases [4].

Recent evidence demonstrates that Web-based diabetes self-management interventions have potential efficacy, feasibility, user acceptability, and uptake [29]. Web programs have demonstrated significant improvements in clinical, behavioral [35-37], psychological, emotional, and psychosocial outcomes [38], as well as a strong uptake and acceptability by both mature [39] and novice users [40].

Recently trialed Web-based diabetes self-management programs based on social cognitive theory (SCT) [41] have demonstrated efficacy [35,42,43]. Central tenets of SCT include self-efficacy, or one’s belief in their capabilities to execute desired courses of action, and outcome expectancies, or personal predictions of likely outcomes resulting from certain courses of action [41]. SCT encompasses the key recommendations in national practice guidelines for diabetes management [44]. These include encouraging patient empowerment (promoting self-efficacy), ongoing monitoring of target outcomes (self-evaluation), and providing diabetes education (instructions). Therefore, SCT provides a theoretical avenue by which to address both behavioral self-management and emotional issues [45]. Self-efficacy has significant positive associations with behavioral outcomes including physical activity participation [46,47], nutrition intake [48,49], weight loss [50], and diabetes self-care [51], as well as with emotional outcomes, including depressive symptoms [52], in people with diabetes.

Despite the growth in supportive evidence on Web-based diabetes programs for self-management, there are some inconsistencies in their results and further data are needed on their potential to improve glycemic control [53]. Lorig et al [54] trialed a diabetes self-management intervention that included modules to assist users in coping with emotional challenges both related and unrelated to diabetes. Although improvements were found in self-efficacy, patient activation, and glycated hemoglobin (HbA_1c_) at 6 months postenrollment, there were no significant improvements in health behaviors, including exercise participation. Emotional outcomes and effects on long-term glycemia were not determined. On the other hand, Glasgow and colleagues [36] reported that their Internet-based diabetes education program (D-Net) did not significantly improve mood or glycemic control, but produced significant improvements in behavioral and psychosocial outcomes. Although current trials show that Web-based diabetes programs have substantial promise, there remains a need to enrich available data on their effects on both mood and glycemia. Given the close mutual influences between dysphoria and diabetes self-management, including the challenging context of psychosocial [55] and emotional stressors [56] in which self-management is attempted, a Web intervention that utilizes a holistic approach to diabetes self-management is required. Such a program would allow users to address both the behavioral and emotional challenges of diabetes in context.

Developing and maintaining commitment to any significant behavior change is challenging. Over the last 30 years, motivational interviewing [57] has demonstrated an ability to enhance motivation, particularly in addictive disorders (where it began), but also in some other behavioral domains [58]. However, until now, its effects on behavior change and glycemic control in diabetes have been inconsistent with only a minority of trials having significant differential effects [59]. Recently, a new approach to eliciting motivation, which uses a motivational interviewing style but trains participants to use motivational imagery in their everyday life, has been advanced [60]. This approach, Functional Imagery Training (FIT), applies 10 years of theoretical and empirical work on the nature and modulation of desires [61]. Personalized multisensory imagery about the benefits of health maintenance behaviors, past successes, and effective strategies is elicited. This is described as television advertisements in which the participant is the actor and is reliving an event or imagining a future event. Participants are asked to practice this imagery when they undertake a routine everyday task and to set electronic reminders for times when they need to undertake a health routine or avoid a dysfunctional behavior. Imagery is cued using photos taken by the participant and is refreshed using new events that illustrate actual gains and effective strategies. Commitment and social support for the goal is elicited by sharing a brief recorded statement about the goal, why it was adopted, how it will be done, and why it will be successful; this recording is replayed if motivation is fading. Brief phone calls by the therapist remind the participant to practice their imagery and help them solve problems by using it.

The study described subsequently comprises a randomized controlled trial (RCT) involving the OnTrack Diabetes program, an automated Web-based intervention aimed to achieve and maintain improvements in type 2 diabetes self-management and dysphoria symptoms. It compares delayed and immediate access to the program, and immediate access supplemented by FIT-based coaching.

## Methods

### The OnTrack Diabetes Program

**Figure 1:**
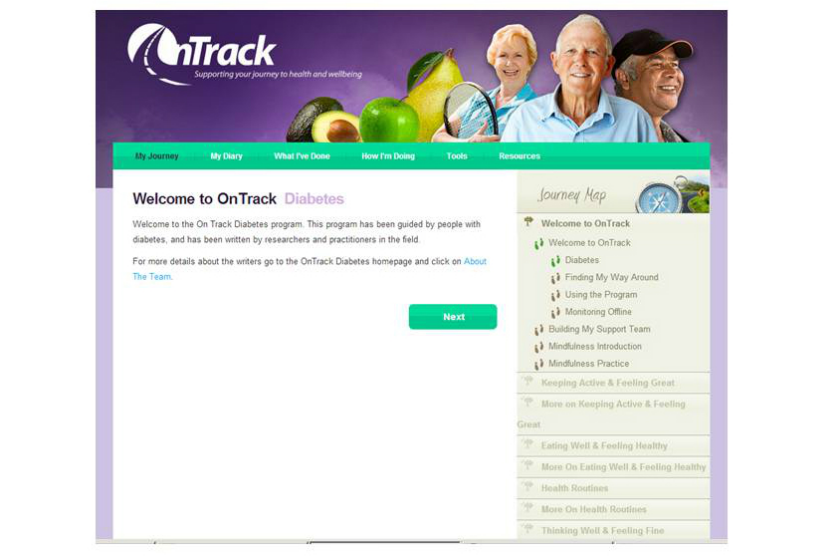
Screenshot of the OnTrack Diabetes home page.

The development of OnTrack Diabetes is described in a companion paper [62](see Figure 1 for screenshot). The program targets physical activity participation, nutrition, adherence to health routines, and mood disturbance (depression, anxiety, and diabetes-related everyday stressors). Specifically, the program includes informational resources: goal setting, planning, and creating routines for self-care behaviors; feeling confident and problem solving via interactive tools; goal attainment scaling via self-monitoring tools, quizzes, and automated feedback graphs; relaxation and mindfulness audios; and an electronic diary. Access to health care providers is also promoted by encouraging users to establish a diabetes care team. Further, the program addresses the independent effects of depression, anxiety, and diabetes-specific distress by enabling users to provide personalized responses in the emotional support tools regarding both diabetes-specific and nonspecific emotional challenges. Although program sections appear in an ordered structure, users can choose to undertake segments in any order—a feature intended to foster user empowerment [63]. Both provided and entered text is minimized with extensive use of icons and pictures and simple sentences and vocabulary so that reading requirements do not exceed Year 7 educational levels.

### Design and Setting

A pilot trial of the program is initially implemented to test for program functionality and to provide an indication of program efficacy, user acceptability, and satisfaction. Following the pilot, a RCT with the participant as the unit of randomization is implemented. The trial is conducted Australia-wide and access is ongoing. The research team is based at the Mitsubishi Centre for Rural and Remote Health at the Wesley Research Institute in Brisbane.

The RCT evaluates the efficacy of the OnTrack Diabetes program in improving the primary outcomes glycemic control (HbA_1c_ level) and diabetes-specific distress symptoms, and the secondary outcomes of depression, anxiety, physical activity participation, diet, blood glucose self-monitoring, and medication taking. User acceptability, ease of use, utility, program satisfaction, and implementation feasibility are also assessed.

**Figure 2:**
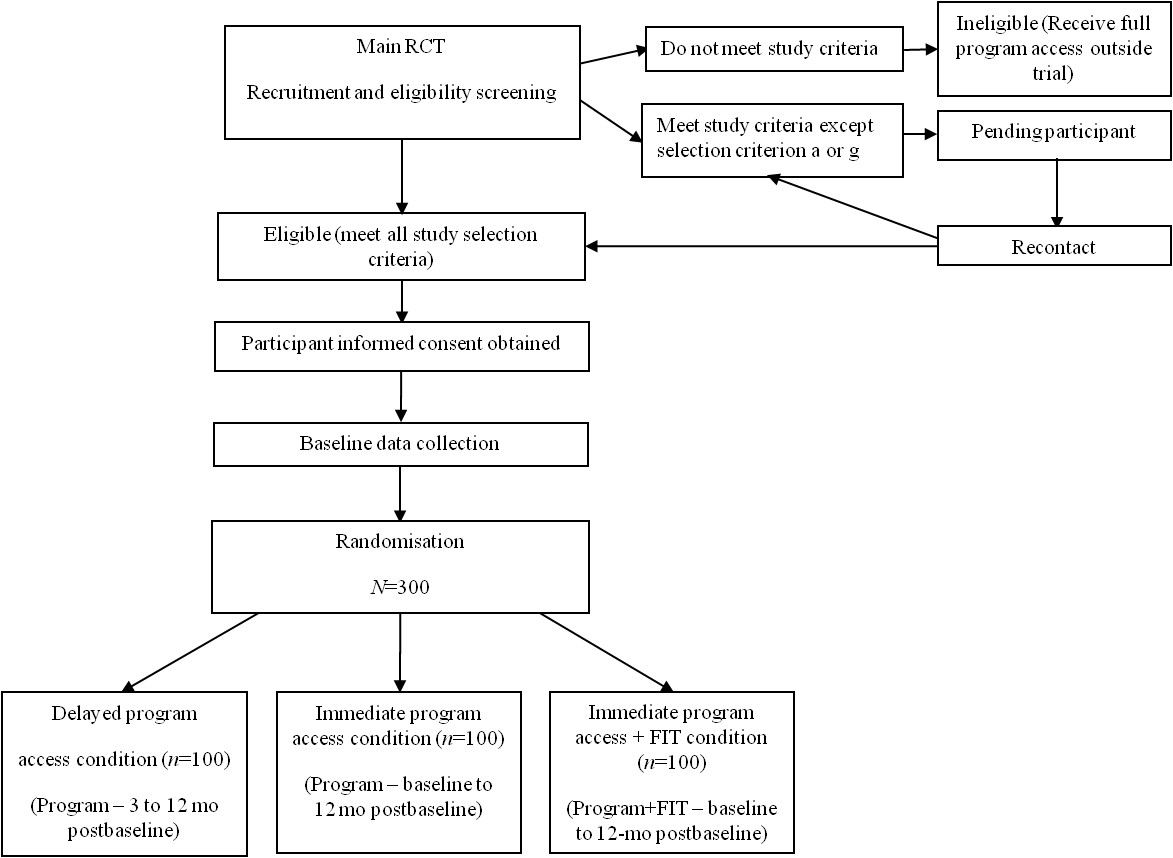
CONSORT flow diagram of the OnTrack Diabetes main randomized controlled trial.

It is hypothesized that at 3 months postbaseline, the immediate access plus FIT condition will show the greatest improvements in primary and secondary outcomes compared with the immediate access and the delayed access conditions. Results of the immediate access and the delayed access conditions are expected to be similar at 6 months when both conditions will have received the full intervention. A CONSORT flow diagram for the trial is shown in Figure 2.

### Recruitment and Sample

Recruitment strategies for the trial are primarily community-based: newspaper advertisements, health organization newsletters, radio broadcasts, notice board postings, bulletins, emails, and online advertisements. Targeted methods include the distribution of study flyers and posters to medical centers, letters to health institutions, pharmacies and health professionals, and featuring the OnTrack Diabetes website URL on statewide Diabetes Australia research Web pages. Attendance at diabetes-related expos and events allows in-person recruitment and further promotion of the project to health professionals.

The trial aims to enroll at least 210 participants. Selection criteria include (1) type 2 diabetes diagnosis (by a medical doctor and according to World Health Organization criteria) of at least 3 months duration, (2) age 18 years or older, (3) living in Australia without plans to leave within 12 months, (4) regular computer and Internet access, (5) contactable by phone, (6) clear command of written English (at least Year 5 education), and (7) stable diabetes pharmacotherapy (medication dose stable ≥4 weeks; medication type stable ≥3 months). Study exclusion criteria include (1) current diagnosis of mental disorder other than depression or anxiety (participant is asked if a condition has been diagnosed and if so they are informed that they will receive access to the program but not be included in the trial), (2) current suicidal risk (assessed via suicide risk assessment), (3) significant cognitive disorder (eg, from head trauma or dementia), (4) currently on steroid medication or likely to commence it in the next 12 months, and (5) pregnant or likely to become pregnant in the next 12 months.

All participants are asked to undertake a medical assessment by their general practitioner prior to study enrollment. Individuals with physical limitations or concurrent physical disorders are advised of the need to exercise caution in setting physical activity goals according with their doctor’s advice.

### Measures

Primary outcomes include diabetes self-care behaviors, HbA_1c_ level, and diabetes-specific distress. Secondary outcomes include depression, anxiety, (medication taking, nutrition intake, physical activity, and blood glucose self-monitoring), quality of life, and user evaluations of the program. Measures are administered at baseline and the 3-, 6-, and 12-month follow-up time points. Table 1 in Multimedia Appendix 1 lists the measures.

### Procedure

Participants register interest on the study website and select a time to undertake an eligibility screening appointment by phone. Eligible individuals undertake baseline measures by email and phone interview at their selected appointment time. Individuals who satisfy all criteria except inclusion criterion time since diagnosis or stable medication are asked if they wish to be recontacted for future screening; if so, they are categorized as “pending.” Individuals who are ineligible are allowed to use the program without being enrolled in the trial.

Following baseline measures, all participants who are randomly allocated to the immediate access or the immediate access plus FIT interventions receive a secure username and password with which to log on to the program. Those enrolled in the delayed access condition are informed that they will receive program access details in 3 months. Computer-generated randomization occurs automatically. One week before the due date for follow-up study measures, participants receive an email notification with a link to the online survey and a preset time for the timeline follow-back procedure phone interview. The email requests that participants email the researcher if the phone interview time does not suit them.

### Delayed Access

In this condition, participants undertake their usual diabetes care with no Web-based program access. Following 3-month follow-up study measures, they receive access to the full OnTrack Diabetes program. Their access to the program is actioned by a member of the research team after which they receive an email that contains an exclusive username and password to access the program.

### Immediate Access

Participants receive access to the full OnTrack Diabetes program from baseline as described in the companion paper about the development of OnTrack Diabetes [62]. This includes access to extensive information resources, a nutrition module (Eating Well and Feeling Healthy), a module focused on adherence to health regimens (Health Routines), an emotional well-being module (Thinking Well and Feeling Fine), and a maintenance module (Keeping On Track). The first section of each module includes a series of interactive tools that incorporate guided imagery, planning goal implementation, goal setting, and confidence building. The planning tools guide participants in proceeding toward their own personalized goals in incremental steps. The second section of each module is entitled “More on...” (eg, More on Health Routines) and involves setting a weekly schedule to increase participation in desired health behaviors and pleasurable activities, and tools for problem-solving challenges. A printable summary is provided on completion of each interactive tool. These summaries can be accessed on future occasions and participants can also complete tools or modules repeatedly if they wish. Self-monitoring tools allow daily monitoring of highest and lowest blood glucose levels, best and worst mood, and the degree that physical activity and nutrition goals were met. Automated feedback graphs on progress are provided for the past week, month, or 3 months. Mindfulness audios are playable on the computer or can be downloaded to an MP3 player.

### Immediate Access Plus Functional Imagery Training

In addition to content received in the immediate access condition, participants in this condition receive regular therapist support phone calls wherein FIT techniques are utilized. The therapist is a provisionally registered psychologist who contacts participants twice in the first week of study enrollment and once in the second and third week of enrollment followed by biweekly calls. Calls last for an average of 30 to 40 minutes until the participant has been enrolled for 1 month, after which they last approximately 20 minutes. Participants are encouraged to practice FIT at home every day. The approach assists them to develop and maintain personalized changes to their diabetes self-care behaviors, alcohol use, smoking, or lifestyle. The therapist reinforces the imagery-based activities already included in the OnTrack Diabetes program and extends these by encouraging additional imagery rehearsal to further enhance motivation.

### Statistical Analyses

Preliminary analyses assess for baseline differences and subsequent analyses control for any observed differences. The primary analyses will comprise multiple regressions, predicting posttreatment and follow-up results from baseline measures and treatment contrasts with multiple imputation being used to predict missing data. Mixed-model ANOVAs with repeated measures will also be applied to confirm whether effects are still obtained without imputations. Both methods allow an intention-to-treat approach to the data. Repeated measures ANOVAs will be used to evaluate differences in change scores between the study time points among the 3 study conditions.

User satisfaction, perceived ease of use, and usefulness are examined with ANOVAs and program reach, acceptability, implementation feasibility, and outreach are assessed using the Reach Effectiveness Adoption Implementation Maintenance (RE-AIM) framework [64]. This will be operationalized using the OnTrack Diabetes Evaluation Questionnaire, which asks about users’ perceptions of the program’s acceptability, issues with feasibility, and outreach (ie, ability to access the program and barriers to access including poor broadband network availability). The inclusion of residents of rural and regional areas enriches this evaluation as will quantitative data about participants’ exposure to the program.

### Sample Size and Retention

The sample of 210 enables detection of a small effect size of *f* ^*2*^=.046 in the multiple regressions. Although the study uses all allocated participants in the outcome analyses, efforts are made to maximize retention by maximizing rapport at the baseline assessment and collecting multiple means to contact participants, including email, landline, and mobile phone contact details, and by assertive follow-up. With these strategies, the study is aimed to have at least 70% retention at 12 months.

### Ethics

Ethics approval to conduct this project was granted by the Uniting Care Health Human Research Ethics Committee (#Cassimatis9111) and the Queensland University of Technology Human Research Ethics Committee (#1100000783).

## Results

This trial is currently underway with funding support from the Wesley Research Institute in Brisbane, Australia.

## Discussion

Results from this trial will provide information on the efficacy, practicality, and user perspectives on the effects of such a program and the success of its dissemination within the Australian context. A 12-month follow-up period will provide data on the maintenance of effects from the programs as well as patterns of usage over time and the relationship of these variables to study outcomes.

Previous trials of diabetes self-management Web-based programs have indicated that a common limitation of such programs is reduced user engagement over time [36,54,64]. It is expected that ongoing access to interactive tools and self-monitoring that users can apply to issues with self-management and dysphoria as they arise will help to maximize engagement and retention. Fortnightly email reminders may also assist users to keep on track of their program usage and prompt continued user engagement with the program [65]. Follow-up assessments during the trial will ask about usage of other treatments and website resources, but the validity of those reports cannot be guaranteed.

Results will provide information about the effectiveness of using a self-guided approach to a Web-based type 2 diabetes self-management intervention. Limitations to generalizability that commonly affect studies conducted within specific clinical or experimental settings are avoided [64] by providing nationwide access and enabling the intervention to be conducted at any location from which access to the Web via a computer is possible. As well as assessing whether the OnTrack Diabetes program will improve type 2 diabetes self-management and dysphoria, this trial is intended to serve as a source of information about the successes and shortcomings entailed in implementing such a program. Results will provide information on which future trials of Web-based cognitive behavioral therapy interventions can build in terms of processes that enhance and those that impede the practicality and rigor of research in this domain.

## Appendix

Multimedia Appendix 1 Measures used in trial.

## Abbreviations

FIT: Functional Imagery Training
RCT: randomized controlled trial
SCT: social cognitive theory
