# Supplementary material for: The OnTrack Diabetes Web-Based Program for Type 2 Diabetes and Dysphoria Self-Management: A Randomized Controlled Trial Protocol
Source: JMIR Res Protoc. 2015 Aug 4;4(3):e97. doi: 10.2196/resprot.2813 (PMC4705366; doi:10.2196/resprot.2813)
Supplement: Multimedia Appendix 1 [file resprot_v4i3e97_app1.pdf]

Measurement area	Outcomes assessed	Measure/s
Demographics	*Age, gender, type 2 diabetes duration, education level, nationality, country of birth, relationship status, employment status, occupation, income, private health insurance*	Short answer and multiple choice items
Clinical	Glycosylated haemoglobin A1c (HbA1c) level	Venous blood sample
Emotional/ Psychological	Depression, anxiety, stress levels	Depression, Anxiety, Stress (brief version; DASS-21) scale (Handley, Shumway, & Schillinger, 2008)
	Diabetes-related emotional burden and interpersonal distress sub-scales	Diabetes Distress Scale (Fisher et al., 2008)
	Self-efficacy for diabetes self-care: blood glucose monitoring, physical activity, nutrition, medication-taking	Diabetes Self-Efficacy Scale (Kavanagh et al., 1993)
	Health-related quality of life	EQ-5D (Shea, 2007)
Behavioral	*Physical activity*	Active Australia Survey (Armstrong, Bauman, & Davies, 2000)<br>OnTrack Diabetes survey
	Dietary intake – number of serves of fruit, vegetables, sweet and fatty foods in the past week	OnTrack Diabetes survey
	Diabetes self-care – blood glucose self-monitoring, medication-taking, nutrition and physical activity	Diabetes Self-Care Activities Survey (Toobert, Hampson, & Glasgow, 2000)
User program evaluations	Users' internet usage, program usage, perceived utility and acceptability, ease of use, user interface, and satisfaction with program	OnTrack Diabetes Program Evaluation Survey

Note: Measures of nutrition intake, demographics, and the OnTrack Evaluation Questionnaire are specific to this study.
